# A simulation-based investigation of the robustness of sequences of operation to zone-level faults in single-duct multi-zone variable air volume air handling unit systems

**DOI:** 10.1177/1420326X241265277

**Published:** 2024-07-20

**Authors:** Narges Torabi, H Burak Gunay, William O'Brien

**Affiliations:** Department of Civil and Environmental Engineering, 6339 Carleton University, Ottawa, ON, Canada

**Keywords:** Fault-tolerant, sequences of operation, ASHRAE Guideline 36, supply air temperature setpoint, duct static pressure setpoint, mode of operation

## Abstract

High-performance sequences of operation for variable air volume (VAV) air handling units (AHUs) respond to requests from zones, leaving these sequences vulnerable to faults that occur at the zone level. While prior research has shown that ASHRAE Guideline 36 reduces energy consumption, its ability to withstand zone-level faults is understudied. This paper investigates the fault tolerance of supply air temperature (SAT) setpoint, duct static pressure (DSP) setpoint and mode of operation (MOP) in single-duct multi-zone VAV AHUs. First, customized sequences were defined for different scenarios in EnergyPlus in this study. Then, common actuator/sensor faults were injected into one zone to identify the most efficient fault-tolerant scenario. The results indicate that trim and respond reset logic is the most fault-tolerant scenario for this paper’s case study, with a 12% energy use intensity (EUI) increase for the SAT setpoint and an 11% increase for the DSP setpoint. Moreover, implementing an average temperature–based control for MOP significantly reduces the setup/setback mode activation, resulting in a lower EUI.

## Introduction

Variable air volume (VAV) air handling unit (AHU) configurations are commonly used in heating, ventilation and air conditioning (HVAC) systems to control the temperature, humidity and airflow in commercial and institutional buildings. However, these systems can be prone to faults due to a variety of reasons, including component degradation (sensors, actuators and control devices can deteriorate over time, leading to failures^1,2^), human error (faults can be caused by human errors during design installation, operation and maintenance of the system^3^) and system complexity (there are many interdependent components that need to work together in a coordinated manner increasing susceptibility to faults and configuration errors^4^). Therefore, fault detection and diagnostic (FDD) tools have been developed to detect, isolate and diagnose faults promptly and effectively.^5^ FDD algorithms integrated into building automation systems (BAS) of VAV AHUs can improve energy efficiency, reduce maintenance costs, increase occupant comfort and extend equipment life.^6^ On the other hand, FDD does not, by itself, eliminate faults and their effects, and it requires the building operators to take action on the identified faults to correct them in order to achieve energy savings.^7^ Identified faults requiring human intervention often result in delay or inaction, leading to increased operations and maintenance costs or degraded comfort conditions.^8^ To solve this problem, fault-tolerant control (FTC) systems are designed to mitigate potential fault effects. An FTC system is a closed-loop control system that can mitigate component malfunctions impact while preserving desirable performance and stability properties.^9^ Depending on the severity of the fault, the use of FTC allows the system to continue operating despite the presence of one or more faults.^7^ Researchers have proposed various FTC methods to preserve overall control performance and minimize any adverse effects on energy consumption or occupants’ comfort caused by system faults.^10,11^

Sequences of operation are the fundamental aspects of VAV AHU control systems regulating temperature, humidity and air quality by employing hardware components such as sensors, actuators and controllers to achieve the desired setpoints. However, they are typically custom-designed based on the specific requirements of the building and usually written in a language specific to the control contractor during installation, without quality control procedures.^12^ So, the energy efficiency of these sequences relies on programmer skills, comprehension of the building systems, commissioning and timely updates for operational changes.^12^ In addition, there is a lack of shared information on specifying the optimal sequences regardless of the programming language used.^12^ Therefore, ASHRAE Guideline 36 (G36)^13^ establishes high-performance sequences of operation for HVAC systems to maximize their energy efficiency. However, these high-performance sequences provided by G36 depend heavily on the number of requests coming from individual zones. This programming approach can make the sequences vulnerable to faults if one or more zones deliver faulty data or requests. Hence, this paper investigates the fault tolerance of sequences of operations commonly used in the building industry (including G36) for multi-zone single-duct VAV AHU systems.

### Background and previous work

Although G36 is a comprehensive reference for high-performance sequences of operation, implementing its sequences often requires complex algorithms, and their improper employment is common, even in less advanced control systems. A few papers investigated implementing G36 sequences of operation using different simulation tools and demonstrated that it could reduce HVAC energy use significantly. For example, Zhang et al.^14^ worked on applying G36 sequences of operation for single-zone VAV AHU systems. They implemented the G36 logic using control description language (CDL) and customized the logic for economizer, supply air temperature (SAT) setpoint reset, fan speed control and zones heating and cooling states. They concluded that the G36 controller saved 17.3% of annual HVAC energy use against the conventional control strategy. Again, Zhang et al.^15^ used EnergyPlus and Modelica to simulate the pressure flow network of an air-based HVAC system and evaluated the HVAC energy consumption using the G36 sequences for multi-zone VAV AHU systems. The simulation results showed an average of 31% savings for the G36 control. Also, they revealed that climate and building operating hours had a greater impact on the energy savings potential than the internal load density. Additionally, Wetter et al.^16,17^ developed a CDL that enables expressing sequences of operations in a digital, machine-readable language. They provided ready-to-use libraries of G36 sequences as well as any user-added sequences that can be configured to a particular building, and the complexity of logic implementation can be hidden from the end user. They showed that with CDL-conforming sequences linked to building energy models, energy savings could be compared for different control strategies. They translated CDL sequences to a commercial control product line using code generation to eliminate programming errors when implementing the control specification. Their work initiated the development of ASHRAE Standard 231P,^18^ which aims to standardize the digital representation of sequences of operation.

In a field study conducted by Nassif,^19^ G36 duct static pressure reset logic was analyzed by testing a multi-zone VAV system in an HVAC lab controlled by a typical commercial BACnet web-based BAS. They measured the performance of various components, such as the supply fan, duct static pressure and VAV boxes, under different testing conditions. Simulation studies were also conducted using EnergyPlus to assess the performance of each component over an extended period. The results of both the field and simulation studies demonstrated that adding the trim booster and respond booster to the traditional trim and respond algorithm can improve the performance of VAV boxes by reducing the time the boxes starved while ensuring that energy savings were obtained for the fan. In another field study performed by Yoon et al.,^20^ a two-storey multi-zone rooftop unit (RTU) VAV system was selected as a test building. The G36 sequences of operation were adopted for the RTU and VAV control to check whether the implemented sequences could communicate with the HVAC system as expected. The SAT and supply airflow rate pattern from each VAV box and discharged air temperature from the RTU were consistent with the expected pattern of G36 control logic.

Also, Lu et al.^21^ compared the G36 sequences of operation to an optimization-based controller (OBC) and a deep reinforcement-learning-based controller (DRLC) in terms of energy efficiency and thermal comfort. A medium-sized office building with a VAV cooling system was simulated in Modelica, and the baseline control system was implemented with the airside and waterside G36 sequences. The optimal SAT and duct static pressure (DSP) setpoints were defined by the optimization problems in OBC and trained control policy by DRLC to minimize the energy consumption and zone air temperature violation. The results demonstrated that the airside sequences of operation in G36 have comparable energy efficiency and thermal comfort performance with two intelligent controllers. Although G36 consumed 7% more energy in the testing shoulder period and has slightly more zone air temperature violations in all testing scenarios compared to the two intelligent controllers, considering the complexity and tuning efforts of the intelligent controllers, it can be concluded that G36 sequences are satisfactory.

The papers mentioned above are limited by investigating the implementation of G36 control logic for single/multi-zone VAV systems either using a field test study or simulation tools. However, the robustness of these sequences to the zone-level faults has not been investigated, and how these sequences handle and adapt to the various types of faults is still largely unknown. The most relevant paper we could find relevant to this subject was research performed by Lu et al.^22^ that investigated a comprehensive fault impact analysis of the G36 sequences. They developed a Modelica-based medium office and followed the airside and plant-side sequences of operation in G36. Different fault scenarios were injected into the baseline model in three different seasonal operating conditions (cooling, shoulder and heating seasons). They evaluated key performance indexes (KPIs) for operational cost, source energy, site energy, control loop quality, thermal comfort, ventilation and power system metrics. The results indicated that different types of faults were identified to be the most influential faults for the majority of the KPIs during different seasons. During the cooling season, severe cooling coil fouling and a stuck cooling coil valve were observed. In the shoulder season, only the stuck cooling coil valve was observed. Finally, during the heating season, the faults with the greatest impact were identified as no temperature setback faults, a stuck heating coil valve and a zone air temperature bias. Ultimately, they concluded that G36 sequences are well adapted for most (∼90%) fault scenarios over all the KPIs.

High-performance sequences of operation in multi-zone VAV AHU systems rely on zone-level sensors and actuators, and the logic is defined based on the number of zones’ requests. Such dependencies make these sequences vulnerable to zone-level sensor and actuator faults. Thus, investigations are needed to ensure that these sequences are robust to common faults at the zone level. As previously mentioned, there are no known existing studies on fault tolerance of high-performance sequences of operation. In order to address the gap identified in the literature, this paper investigates the fault tolerance of customized sequences of operation for single-duct multi-zone VAV AHU systems using a building performance simulation (BPS) tool.

### Scope of work

The focus of this paper was investigating the robustness of sequences of operation for the airside of HVAC systems, specifically single-duct multi-zone VAV AHU systems. The most vulnerable high-performance sequences of operation to the zone-level faults for such systems are SAT and DSP setpoint reset logic.^23^ For example, placing a thermostat close to heat-generating equipment in one of the zones causes the AHU to cool down this constantly warm zone by resetting the SAT setpoint to lower values. This temperature bias has to be compensated for by the reheat coils of all other zones, resulting in energy waste. Correspondingly, a stuck VAV damper in the fully open position in one of the zones causes the AHU to provide more airflow to that constantly starving zone by resetting the DSP setpoint to higher values, increasing fan electricity use. In addition to SAT and DSP setpoint reset, the AHU mode of operation (MOP) is another control logic that can be negatively affected by a faulty zone. For instance, a stuck closed radiator valve in a zone can disrupt the AHU night cycle and wake the AHU up by changing its mode from unoccupied to a setback. Therefore, such dependencies make it necessary to investigate the robustness of these sequences to the sensor and actuator faults at the zone level. In this paper, we studied the fault tolerance of SAT setpoint, DSP setpoint and mode of operation logic. We defined the customized sequences of operation for different control scenarios in the energy management system (EMS) application of the building performance simulation (BPS) tool EnergyPlus. The HVAC energy consumption for different scenarios in the presence of faults was compared to see which scenario is robust enough to absorb the fault at the zone level and maintain satisfactory performance of the system. In other words, the fault-tolerant control logic is able to identify the rogue zones and disregard requests coming from these zones to the AHU controller.

## Methodology

Different SAT and DSP setpoint reset scenarios commonly used in the building industry or proposed by researchers are defined in the EMS, a programming environment that enables the implementation of customized control logic in EnergyPlus. For the mode of operation, different temperature-based control logics are defined in the EMS to start and stop the AHU night cycle. Details of each scenario for all three sequences (SAT, DSP and MOP), the type of faults that are applied manually into one of the zones, and how the faulty zones are identified and the requests from them are disregarded are explained in the rest of this section. Finally, the section concludes by discussing a case study that was modelled in EnergyPlus to examine the energy consumption of each scenario in the presence of faults.

### SAT setpoint scenarios

#### Constant

In the first scenario, SAT was set to 12.8°C throughout the year in this study. This is a traditional practice to satisfy the maximum cooling load. However, this strategy leads to an increase in the reheat load and cooling compared to resetting the SAT setpoint.^24^ Due to the lower SAT during periods of low cooling load, additional heating may be needed to maintain the desired indoor temperature. This additional heating that has to be provided by the reheat coils increases the energy consumption of the HVAC system.

#### Outdoor air temperature reset

**Figure 1.**
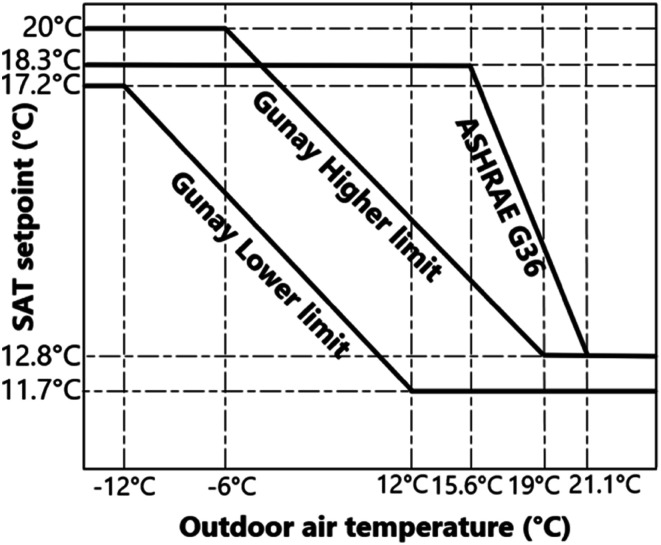
OAT reset scenarios: G36,^13^ Gunay et al.^25^ higher limit for low-density occupancy and Gunay et al.^25^ lower limit for high-density occupancy.

Although G36 does not endorse using OAT reset logic solely and recommends the hybrid reset strategy, an SAT setpoint reset strategy based on outdoor air dry-bulb temperature, which is proposed in G36 as an example, was selected in this study and is shown in Figure 1. Note that higher and lower OAT values are climate-dependent and are defined according to the project location and designer/engineer expertise. In addition to the example from G36 used in this paper, researchers studied the effect of OAT on SAT setpoint and proposed different OAT-based SAT setpoint reset profiles in several papers.^25–27^ Amongst them, the higher and lower optimal reset profiles proposed by Gunay et al.^25^ based on low and high occupancy levels have a more extensive range encompassing other profiles. Gunay et al.^25^ Optimized the SAT setpoint for 27 variants of the generic EnergyPlus office building model representing three levels of occupancy, envelope and HVAC capacity scenarios. Amongst those three levels investigated, occupant density had the most significant impact on the optimization results. Therefore, with the low-density occupancy scenarios, they recommended providing SAT as high as 20°C when the OAT was less than −5°C. In contrast, with high-density occupancy scenarios, they recommended the optimal SAT setpoint not to exceed 16°C, even when the OAT was as low as −20°C. In this paper, we implement three OAT-based SAT setpoint resets: OAT reset proposed by G36, OAT reset for low-density occupancy (higher limit) and high-density occupancy (lower limit) proposed by Gunay et al.^25^ (as shown in Figure 1). Note that the OAT-based reset strategy saves reheat load and increases the benefit of an airside economizer. However, it does not account for the cooling demands of individual zones.^28^ Consequently, some zones (e.g. conference rooms, core zones or highly glazed south-facing zones) may require a large amount of cooling, regardless of the OAT, and their cooling demands are not considered while resetting the SAT setpoint.

#### Trim and respond reset

Unlike dual-duct VAV systems that both heating and cooling requests are considered in the control loop, in single-duct multi-zone VAV AHU systems, the control of the SAT setpoint is driven by cooling demand to ensure that the most demanding cooling loads can be met; thus, only zone cooling requests are defined in the T&R logic discussed in this paper. In G36, the T&R logic resets a setpoint for SAT by increasing the setpoint at a fixed rate until a downstream zone is no longer satisfied and generates a cooling request. When sufficient requests are present, the SAT setpoint is decreased in response. When sufficient cooling requests no longer exist, the setpoint resumes increasing at its fixed rate. Note that the importance multiplier factor can adjust the importance of each zone’s requests to ensure critical zones are always satisfied. In other words, each zone can be assigned an importance multiplier greater than one to increase the rank of that specific zone. However, in this study, the importance multiplier of 1 (the default number) was assumed for all zones in the model. The T&R variables used in the EMS model are listed in Table 1. G36 does not endorse using T&R reset logic solely and recommends the hybrid reset strategy. However, the reason for including OAT reset and T&R reset in this study is that control engineers and BAS integrators continue to utilize them when designing and implementing sequences of operation within the building industry.

**Table 1.** T&R variables for SAT setpoint reset.

Characteristics	Content
Minimum SAT setpoint	12.8°C
Maximum SAT setpoint	18.3°C
Initial SAT setpoint	Maximum SAT setpoint
Time interval	2 min
Trim amount	0.1°C
Respond amount	−0.2°C
Maximum response per time interval	−0.6°C
Number of ignored requests	2°C
Number of requests	3 requests if: zone temperature > zone cooling setpoint +3°C
	2 requests if: zone temperature > zone cooling setpoint + 2°C
	1 request if: zone temperature > zone cooling setpoint + 1°C

The reset logic was started with the initial SAT setpoint when the AHU supply fan was turned on. Then, the setpoint was increased in each time step (5 min) by the trim amount (0.1°C). If the sum of cooling requests from all zones exceeded two, the setpoint was decreased by the corresponding response amount multiplied by the difference between the number of requests and the number of ignored requests (2) but no more than the maximum response per time interval (−0.6°C). In other words, the SAT setpoint was reset within the minimum and maximum setpoint and was calculated using the SAT setpoint in the previous time step plus the trim/respond amount.

#### Hybrid reset

**Figure 2.**
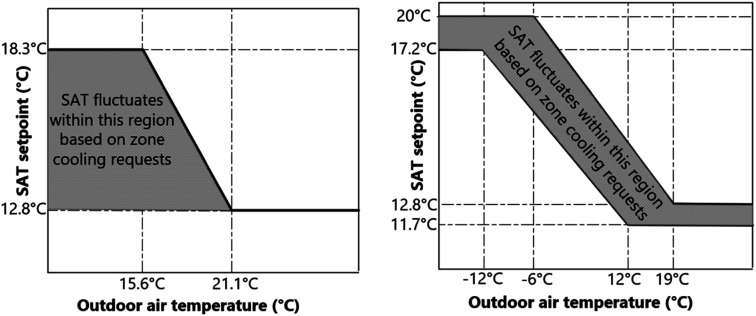
Hybrid reset scenarios, left: G36^13^ and right: Gunay et al.^25^

The SAT setpoint reset during occupied mode is recommended by G36 to be a mix of OAT reset and zone feedback. This approach aims to reduce fan energy during warm weather and satisfy the cooling setpoint for the warmest zone. As illustrated in Figure 2, a hybrid reset control incorporates and combines both open-loop (OAT reset) and closed-loop (T&R reset) control systems. In this study, we investigated two hybrid reset scenarios; one was a combination of G36 OAT and T&R reset, and the other was a combination of Gunay et al.’s^25^ higher and lower limits, and G36 T&R reset logic.

### DSP setpoint scenarios

#### Constant

The traditional practice is to set the DSP to a fixed number. In this strategy, fan speed is adjusted to maintain a constant static pressure level under every operational condition, which provides excessive static pressure during partial load, resulting in increasing fan power consumption and air leakage in the ductwork.^29,30^ In this study, for the first scenario, the static pressure rise experienced by the supply fan was set to 300 Pa.

#### Critical zone reset

ASHRAE 90.1^31^ for systems with direct digital control of individual zone boxes reporting to the central control panel recommends resetting static pressure setpoint based on the zone requiring the most pressure, that is, the setpoint was reset lower until one zone damper is nearly wide open. In the critical zone reset strategy, the duct static pressure was reset to maintain the critical zone damper wide open at any load conditions during operation.^32^ In this scenario, the control system monitored the operation of each VAV terminal to identify the zone with the most demanding damper position. It then unloaded the supply fan just enough to keep the VAV damper in the critical zone fully open.^33^ However, 90% was used here as a fully open damper position rather than 100% since a PID loop requires an error from the setpoint on both sides of the setpoint.^34^ So, in the EMS model, when the critical zone’s damper was less than 90% open, the pressure rise at the fan was decreased at a fixed rate until the damper position was above 90%, where the pressure rise was increased at the same fixed rate. Although unloading the fan in this fashion to not generate more pressure than required to satisfy the critical zone reduces the fan’s energy consumption and noise level, a fault in one of the zones may cause that zone to be mistakenly recognized as a critical zone and disrupt the DSP setpoint reset.

#### T&R reset

In G36, T&R logic resets a setpoint for DSP by decreasing the setpoint at a fixed rate (trim amount) until a downstream zone is no longer satisfied and generates a request. The requests are generated based on the measured airflow and VAV damper position. The number of requests from all zones is summed up, and when a sufficient number of requests are present (the number of requests exceeds the number of ignored requests), the DSP setpoint is increased in response with a specified rate (respond amount). When enough requests are no longer present, the setpoint resumes decreasing at its fixed rate. The T&R variables for the DSP setpoint reset used in the EMS model are listed in Table 2. Although EnergyPlus has certain inherent limitations for pressure reset, it remains a versatile research custom program with an integrated and comprehensive EMS to implement customized sequences of operation. We overcame this limitation, successfully implementing a pressure reset by utilizing a pressure rise as an actuator within the EMS and connecting it to control the AHU’s supply fan.

**Table 2.** T&R variables for DSP setpoint reset.

Characteristics	Content
Minimum fan pressure rise	25 Pa
Maximum fan pressure rise	250 Pa
Initial fan pressure rise	120 Pa
Time interval	2 min
Trim amount	−12 Pa
Respond amount	15 Pa
Maximum response per time interval	32 Pa
Number of ignored requests	2
Number of requests	3 requests if: measured airflow <50% of setpoint and damper position >95%
	2 requests if: measured airflow <70% of setpoint and damper position >95%
	1 request if: damper position >95% until the damper position <85%
	0 request if: damper position <95%

### MOP scenarios

G36 classifies seven modes of operation for an AHU: occupied mode, warm-up mode, cool-down mode, setback mode, setup mode, freeze protection setback mode and unoccupied mode. Setback and setup modes are used to keep zone temperatures from staying excessively far from occupied setpoints so that the cool-down and warm-up modes can achieve the setpoint when initiated.^13^ However, as setback and setup modes depend on zone temperature, they are vulnerable to zone-level faults. Consequently, a faulty zone can negatively affect the AHU’s performance by activating the night cycle and cause the AHU to enter the setback/setup mode frequently when the building is unoccupied. In this study, we investigated three temperature-based control scenarios that are commonly used in the building industry to activate the AHU night cycle during the unoccupied mode.

#### G36 temperature–based control

G36 describes entering the AHU mode from unoccupied to setup/setback mode as given below, which was used in this study as the first scenario to investigate the energy use in MOP logic in the presence of faults.

• Setback – If any five zones in the zone group fall below their unoccupied heating setpoints or if the average zone temperature of the zone group falls below the average unoccupied heating setpoint, the zone group shall enter setback mode until all spaces in the zone group are 1°C above their unoccupied setpoints.
• Setup – If any five zones in the zone group fall above their unoccupied cooling setpoints or if the average zone temperature of the zone group falls above the average unoccupied cooling setpoint, the zone group shall enter setup mode until all spaces in the zone group are 1°C below their unoccupied setpoints.

#### Minimum/maximum temperature–based control

In the first scenario, the minimum/maximum zone temperature amongst all zones was used to activate the night cycle. So, if the minimum temperature was below the unoccupied heating setpoint (15.6°C) minus temperature offset (0.5°C here), then the night cycle would activate in the EMS by overriding the air system to start when it would normally be off, and the AHU entered setback mode. Similarly, if the maximum zone temperature amongst all zones was above the unoccupied cooling setpoint (30°C) plus temperature offset (0.5°C), then the AHU entered the setup mode. The zone temperatures were checked in each time step (5 min here) to ensure the AHU was turned on and off properly based on the difference between minimum/maximum temperatures amongst all zones and zone unoccupied heating/cooling setpoints.

#### Average temperature–based control

In this scenario, the average temperature of all zones was utilized for comparison against the zone unoccupied setpoint to activate the night cycle and change the AHU mode from unoccupied to setback/setup modes. Assuming unoccupied heating setpoint of 15.6°C and cooling setpoint of 30°C, a temperature offset of 0.5°C and a timestep of 5 min, the logic employed in this scenario was similar to the previous one except for the utilization of the average temperature across all zones instead of the maximum or minimum temperature.

### Rogue zones

A rogue zone is defined in this paper as a zone that always sends cooling requests based on SAT setpoint reset logic, always sends pressure-increase requests based on DSP setpoint reset logic, or constantly changes the AHU mode from unoccupied to setback/setup in the mode of operation logic. These cooling/pressure requests or AHU mode changes were generated due to a zone-level fault either caused by a physical failure (e.g. broken damper/valve) or a human error in the design/installation phase (e.g. improper thermostat location).

#### Identifying rogue zones

An ultimate T&R reset logic needs to identify the rogue zone and lock it out from the control loop. As per G36, we identified rogue zones when the zone cumulative %-request-hours exceeded 70% and the total number of hours the zone/systems have been operating exceeded 40 h. The cumulative %-request-hours are defined as the zone request hours divided by the system run hours (the hours in any mode except unoccupied mode). After identifying the rogue zone, the important multiplier was set to zero for that zone, and the requests originating from the zone to the AHU controller were not taken into account. Finally, when the number of hours the system has been operating in any mode other than unoccupied mode exceeded 400 h, the important multiplier was reset to one for all the zones, the system run hours were reset to zero, and the reset logic was started from the beginning. In fact, if a zone sent requests more than the specified threshold for the first 40 operating hours, the requests would be temporarily disregarded for a duration of up to 400 h while the AHU continues to operate. After this 400-hour period, the reset was implemented for all zones, including the previously identified rogue zone, and the requests from that zone were then taken into consideration. An alarm was also generated to notify building operators when a rogue zone was identified. G36 classifies the different levels of alarms based on delay time, suppression periods and whether they require acknowledgement from operators before they can return to normal.

In addition to the alarm for a rogue zone identified in T&R logic, G36 introduces high-temperature and low-temperature alarms for when a zone temperature is above/below the cooling/heating setpoint for 10 min by 2°C or 3°C (based on the alarm level). However, as stated by G36, zone temperature alarms are not suppressed in setup, setback or unoccupied modes to ensure heating or cooling equipment or control failures are promptly detected. Failure to identify such issues could result in excessive pull-down or pick-up loads and even freezing of pipes.^13^ Therefore, in this study, we did not eliminate rogue zones from the control loop in MOP logic. Instead, they were identified by a zone temperature alarm that could not be suppressed unless the building operator fixed the issue.

#### Generating rogue zones in the model

To generate a rogue zone in the model, we manually apply the most common faults with opposite impacts on each logic in one of the perimeter zones. The faults integrated into a zone for different SAT setpoints scenarios are: offset thermostat to 5°C higher than the room’s actual temperature by implementing operational faults (fault model: offset thermostat) in EnergyPlus and stuck open VAV damper by increasing the minimum airflow fraction from 20% to 100%. The offset thermostat causes overheating in the zone and generates cooling requests, while a stuck open VAV damper does not generate any cooling requests. The faults integrated into one of the zones for different DSP setpoints scenarios are: stuck VAV damper in the fully open position, which makes the zone appear starving, constantly asking for pressure increase and stuck closed VAV damper (by setting the constant minimum air flow fraction to zero), which makes the zone appear as a satisfied zone in terms of airflow and does not result in generating pressure-increase requests. Finally, the faults injected into one of the zones for MOP logic are: stuck closed radiator valve causing the AHU mode to change from unoccupied to setback mode to heat the zone and offset thermostat to 5°C higher, which drives the AHU mode to enter setup mode during unoccupied hours to cool the affected zone. Table 3 summarizes the scenarios used in this paper for each sequence of operation and the faults generated in the model for that specific sequence to create a rogue zone. Note that the offset thermostat to a higher temperature in EnergyPlus simulation models a common fault scenario where a thermostat is improperly placed either in direct sunlight or near heat-generating equipment. The extent of the error resulting from this placement can vary depending on factors such as the intensity of sunlight, the temperature of the heat source and the sensitivity of the thermostat. Consequently, the thermostat may read higher temperatures than the actual ambient temperature. As an extreme example, we selected a 5°C offset to underscore the notable disparity in energy consumption between a fault-free scenario and one involving an offset thermostat when cooling requests were activated prominently (while studying different SAT setpoint scenarios) and when enabling the AHU setup mode (while studying different temperature-based scenarios in MOP). Also, in this study, the DSP setpoint held constant when investigating different SAT setpoint scenarios. Similarly, the SAT setpoint was kept constant when studying DSP scenarios, and finally, both SAT and DSP setpoints were held constant when examining MOP scenarios. Consequently, if the minimum VAV damper position in one of the zones was manually set to 100% in the EnergyPlus model while the DSP setpoint remained constant during the investigation of SAT setpoint scenarios, this fault had resulted in overcooling as the damper was stuck in 100% position during operational hours while the DSP setpoint was constant and able to meet the zone demanding airflow. This situation differed from a VAV damper being stuck in a fully open position in a starving zone because of a low DSP setpoint.

**Table 3.** Summary of the scenarios and injected faults employed in the model for each sequence of operation.

Sequences of operation	Scenarios		Type of injected faults
SAT setpoint	Constant setpoint		
	G36 OAT reset		
	OAT reset proposed by Gunay et al	Higher limit	• Offset thermostat to 5°C higher than room temperature
		Lower limit	• Stuck open VAV damper
	G36 T&R disregarding requests from rogue zone		
	G36 hybrid disregarding requests from rogue zone		
	Gunay et al.^25^ hybrid disregarding requests from rogue zone		
DSP setpoint	Constant setpoint		• Stuck open VAV damper
	Critical zone reset		• Stuck closed VAV damper
	G36 T&R disregarding requests from rogue zone		
MOP	G36 temperature–based control		• Stuck closed radiator valve
	Min/max temperature–based control		
	Average temperature–based control		• Offset thermostat to 5°C higher than room temperature

The objective of this paper is not to optimize energy consumption but rather to understand how different faults impact the system’s performance. To do this, we conducted a local sensitivity parametric study by introducing faults into the EnergyPlus simulation and observing their impact on energy consumption. However, we only analyzed the impact of individual faults, not exploring how combinations of faults and interactions between parameters influence the system’s performance. Our study concentrated on analyzing how sensitive the model’s output was to a specific common fault to investigate the fault tolerance of sequences of operation. Therefore, we were not exploring how variations in multiple parameters could collectively affect energy consumption (global sensitivity study).

After generating the rogue zones, the HVAC energy consumption in the presence of faults was investigated for each scenario to determine the most fault-tolerant scenario.

### Case study

**Figure 3.**
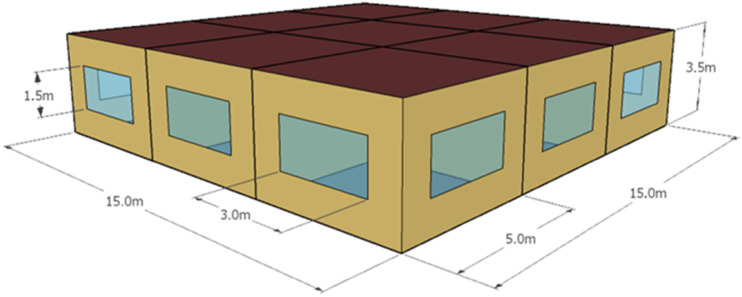
Geometry of EnergyPlus model.

An office building with eight perimeter zones and one core zone located in Ottawa, Canada, with a floor area of 225 m^*2*^ and a window-to-wall ratio (WWR) of 0.26, was created in EnergyPlus, as shown in Figure 3. The climate data used in the simulation model was sourced from the Actual Meteorological Year (AMY) weather files. AMY files capture the precise climatic conditions during extreme weather events, enabling accurate simulation of such scenarios. These weather files provide valuable data that can be used to assess the vulnerabilities and resilience of buildings to extreme weather events.^35^

The heat transfer to the floor above and below was neglected as we assumed the model was between two identical floors. A dedicated AHU, including variable speed supply and return fans, heating and cooling coils, an enthalpy-based economizer and an electric steam-generated humidifier, serves the zones. The AHU heating coil and humidifier are available from October to April, and the cooling coil from May to September. The air is distributed through a single-duct VAV system into the zones. All VAV terminal units are equipped with reheat coils, and all zones except the core zone have perimeter heating devices. The reheat coils are only available during the AHU operating hours, while the baseboard heaters are operational throughout the heating season. The zone setpoint temperatures during occupancy are 22°C and 23.5°C (as per a BPS-based study performed by Gunay et al.^36^ to find the optimal heating and cooling season temperature setpoint), and in the unoccupied mode, were assumed to be 15.6°C and 30°C for the heating and cooling seasons, respectively. The minimum required ventilation rate was calculated as the summation of 2.5 L/s per person and 0.3 L/s per square metre as per ASHRAE Standard 62.1^37^ with the zone distribution effectiveness of one. The AHU is operational on weekdays during occupancy hours. Occupants were assumed to arrive at 7 a.m., and the building would be fully occupied from 10 a.m. until 3 p.m.; then occupants would start departing the building, and the building would be unoccupied at 6 p.m. A constant 3-hour morning warm-up/cool-down is considered for the AHU. The lighting and plug load intensity is linked to the occupant density during occupancy with after-hours lighting ratio and plug load when the building is unoccupied. The EnergyPlus model and EMS codes are provided in the following GitHub link: https://github.com/CarletonDBOM/fault-tolerant_sequences_of_operation.git

## Results and discussions

### Results for SAT setpoint scenarios

**Figure 4.**
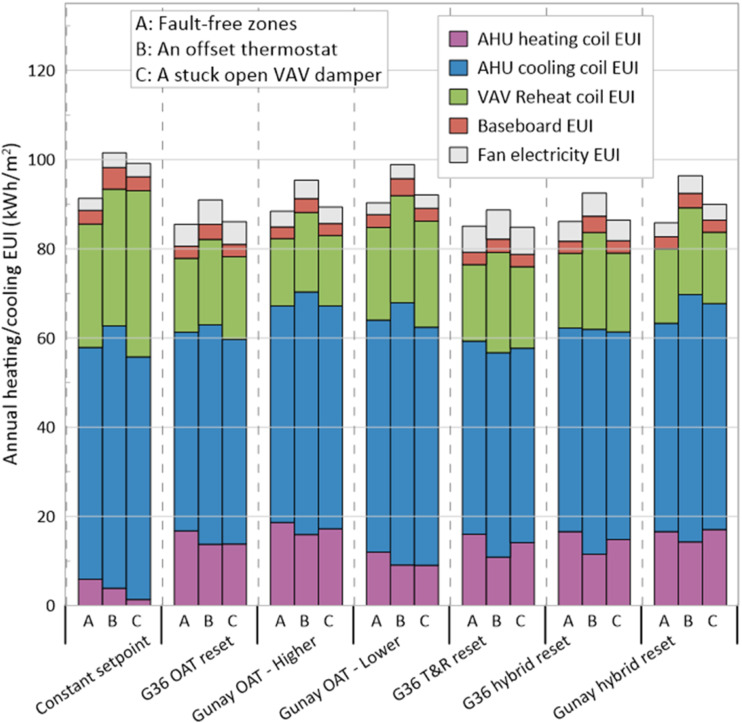
Annual heating and cooling EUI for different SAT setpoint scenarios in the presence of faults.

The annual heating and cooling energy use intensity (EUI) of the model for different SAT setpoint scenarios are shown in Figure 4. These scenarios included situations where the zones are free of faults, a zone with an offset thermostat 5°C higher than the actual room temperature (adjusting the measured temperature to 5°C higher) and a zone with a stuck VAV damper in the fully open position. The figure demonstrates that the T&R reset logic has the minimum and the constant SAT setpoint has the maximum annual EUI for the fault-free situation. Also, as expected, the continuous 12.8°C SAT setpoint has the minimum AHU heating coil EUI and the maximum reheat coil EUI compared to other scenarios for all three situations. In the presence of an offset thermostat, which causes overheating in the zone, the T&R reset logic (capable of identifying the rogue zone) has the minimum annual EUI and is the most energy-efficient strategy. Similarly, the results of a stuck open VAV damper causing overcooling in the zone demonstrate the most energy-efficient strategy is the T&R reset logic. When the SAT setpoint is constant, a stuck open VAV damper results in a notable increase in VAV reheat coil EUI compared to a fault-free situation. However, when employing a reset setpoint, such as an OAT reset, T&R reset or a hybrid reset, the EUI of the reheat coil does not undergo a substantial increase in a stuck open VAV situation. This is because the SAT setpoint is dynamically adjusted based on factors like the OAT and cooling requests, and a stuck open VAV damper does not trigger additional cooling requests.

**Figure 5.**
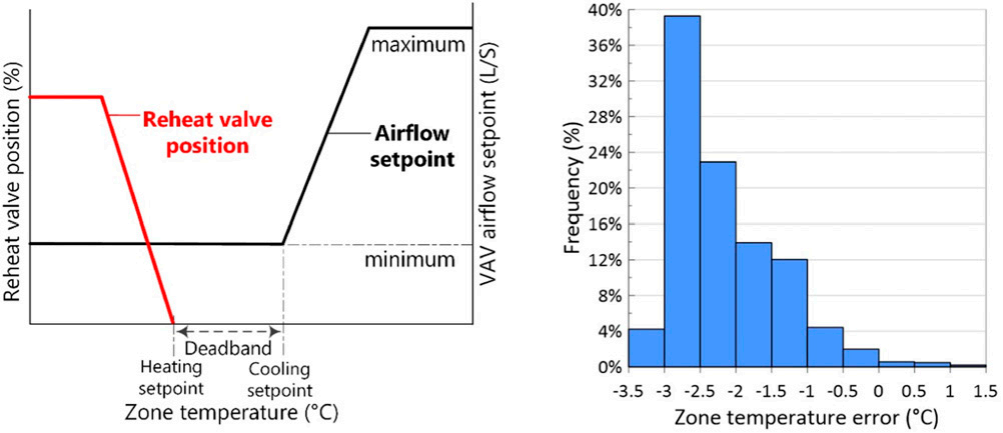
Left: control logic for single-duct VAV systems, and right: distribution of zone temperature error in cooling mode in the presence of a stuck open VAV damper.

In addition to finding the most efficient scenario, we aimed to assess their fault tolerance. To evaluate the fault tolerance of each scenario, the EUI change from fault-free zones to a faulty zone situation was calculated and is shown in Table 4. As shown in the table, the T&R reset logic capable of identifying the rogue zones has the minimum EUI change in the presence of an offset thermostat, so it is the most fault-tolerant SAT setpoint scenario. However, in the presence of a stuck VAV damper in the fully open position, the change in EUI is minimal for all scenarios. The reason is that single-duct multi-zone VAV systems are primarily intended for cooling, and situations like a stuck open VAV damper, which does not generate heating requests, do not disrupt the SAT setpoint reset logic. Two control options exist for the damper controlling the airflow in the VAV during heating operation in EnergyPlus. With the Normal (the default) action used in this study, the damper would remain at the minimum air flow rate during the heating operation. As the heating load was increased, the water flow rate in the reheat coil would be increased to maintain the temperature in the zone until the maximum water flow rate was reached or the user-specified maximum reheat air temperature was reached, as illustrated in Figure 5 (left side). So, with a stuck open VAV damper, the zone is not overheated enough to send cooling requests. However, in the cooling mode, the airflow varies between the minimum and maximum airflow setpoint to meet the zone temperature setpoint.

**Table 4.** Change in annual heating and cooling EUI for different SAT setpoint scenarios in the presence of faults.

SAT scenarios		Change in EUI	
		Offset thermostat (%)	Stuck open VAV damper (%)
Constant setpoint		11	5
G36 OAT reset		6	1
OAT reset proposed by Gunay et al.	Higher limit	7.5	1
	Lower limit	9	2
G36 T&R reset disregarding requests from rogue zone		4	0
G36 hybrid reset disregarding requests from rogue zone		7	0
Gunay et al. hybrid reset disregarding requests from rogue zone		8	1

Thus, since a stuck VAV damper in the fully open position does not generate cooling requests in the zone, it does not significantly change energy use compared to when the zones are fault-free. However, as shown in Figure 5 (right side), it causes overcooling and occupants’ discomfort in the zone. The zone temperature error stated in Figure 5 is the difference between the measured zone temperature and setpoint zone temperature. Therefore, although a stuck open VAV damper does not cause the zone to be identified as a rogue zone based on SAT setpoint reset logic, and the increase it causes in the annual heating and cooling EUI is minimal, it still causes discomfort in the zone during the cooling mode.

### Results for DSP setpoint scenarios

**Figure 6.**
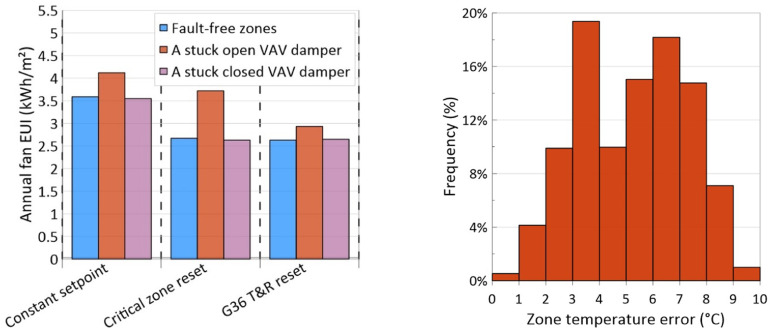
Left: annual fan EUI for different DSP setpoint scenarios in the presence of faults, and right: distribution of zone temperature error in cooling mode in the presence of a stuck closed VAV damper.

The annual fan EUI of the model for when the zones are fault-free, a zone with a stuck open VAV damper and a zone with a stuck closed VAV damper is shown in Figure 6 (left side).

These faults were selected for DSP setpoint logic because a stuck VAV damper in the fully open position could cause the zone to appear as a starving zone and constantly demand an increase in pressure, while a VAV damper that is stuck closed causes the zone to appear as a well-fed zone. As demonstrated in the figure, the constant DSP setpoint has the maximum EUI and the G36 T&R reset capable of disregarding requests from the rogue zone has the minimum EUI and is the most efficient scenario for all three situations. Moreover, a stuck VAV damper in the fully open position would increase the fan EUI significantly in the critical zone reset scenario. This occurs because the damper position reaches above 90%, making the zone a critical zone that continually demands fan pressure rise, which consequently increases fan EUI.

Similar to the SAT setpoint logic, the EUI change from fault-free zones to a faulty zone situation was calculated and is shown in Table 5 to find the most fault-tolerant scenario. As shown in the table, G36 T&R has the minimum change in fan EUI in the presence of a stuck open VAV damper and is known as the most fault-tolerant scenario. When a stuck closed VAV damper fault is injected into one of the zones, the fan EUI change is almost zero for all three scenarios. The reason is a stuck closed VAV damper appears as a well-fed zone that is not identified as a critical or rogue zone and does not generate any pressure rise request. However, it causes overheating in the zone and thermal discomfort during the cooling mode, as shown in Figure 6 (right side).

**Table 5.** Change in annual fan EUI for different DSP setpoint scenarios in the presence of faults.

DSP scenarios	Change in EUI	
	Stuck open VAV damper (%)	Stuck closed VAV damper (%)
Constant setpoint	15	0
Critical zone reset	39	0
G36 T&R disregarding requests from rogue zone	11	0

If a proper T&R reset is put for the SAT setpoint, then the zone with the stuck closed VAV damper would be identified as a rogue zone in the SAT setpoint control loop (as it causes overheating in the zone and constantly sends cooling requests). While the zone with a stuck closed VAV damper is not identified as a rogue zone in the DSP setpoint loop, a high-temperature alarm (according to G36) is activated when the zone temperature is above the cooling setpoint. This alarm notifies the building operators to fix the issue causing thermal discomfort in the zone.

### Results for MOP scenarios

**Figure 7.**
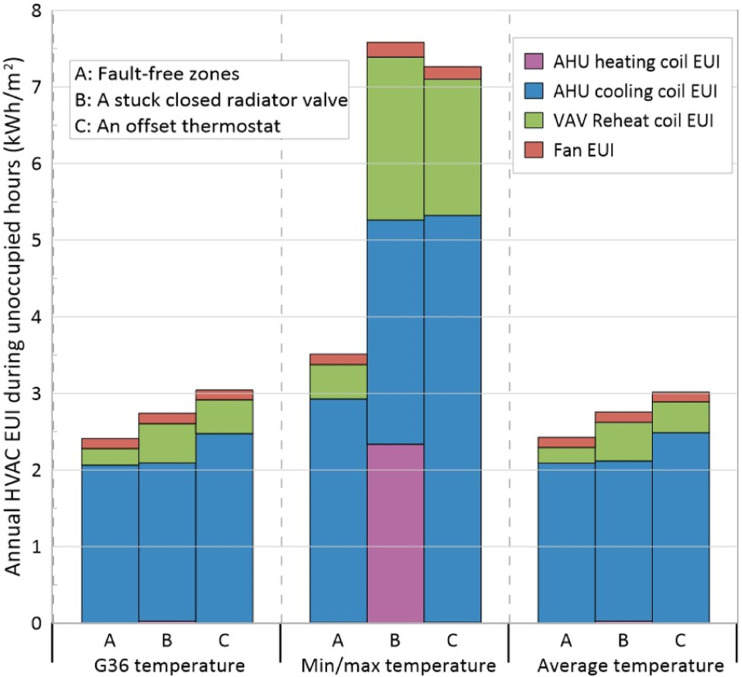
Annual HVAC EUI for different temperature-based MOP scenarios during unoccupied hours in the presence of faults.

The annual HVAC EUI of the model for three situations: fault-free zones, a zone with a stuck closed radiator valve and a zone with an offset thermostat 5°C higher than the actual room temperature, are shown in Figure 7. The EUIs were calculated during unoccupied hours, as activating the AHU night cycle and entering the AHU mode to setback/setup occurs during unoccupied hours. These particular faults were selected because a stuck closed radiator valve changes the MOP from unoccupied to setback during the heating season, while an offset thermostat was set to higher values, which would change the MOP from unoccupied to setup during the cooling season.

The figure demonstrates that the annual EUI during unoccupied hours is almost identical for G36 and average temperature–based control scenarios in all three situations. However, the min/max temperature–based control scenario enables the night cycle more frequently, and as a result, the annual EUI was almost twice as high as the EUI for the other two scenarios in the presence of faults. For example, in a stuck closed radiator valve situation enabling the setback mode, the AHU heating coil EUI was minimal in the G36 and average temperature–based control scenarios, while it was increased significantly when the minimum temperature–based control scenario was employed in the MOP logic. When a radiator valve remained closed in one of the zones while using the min/max temperature–based scenario, the AHU operated more frequently during unoccupied hours. As a result, Figure 7 illustrates an uptick in the AHU heating load. It is important to note that reporting baseboard heating load does not significantly impact the investigation, as the decreased energy in one zone due to a stuck closed radiator valve is overshadowed by the more significant energy consumption of an operational AHU heating coil. Similarly, with an offset thermostat to higher values enabling setup mode, the AHU cooling EUI would increase considerably in the maximum temperature–based control scenario compared with two other scenarios. Consequently, the average temperature–based control scenario is the most fault-tolerant scenario. However, the G36 temperature–based scenario is almost as robust as the average temperature–based scenario.

## Limitations and future work

The EnergyPlus model used in this paper is a simple office space with eight perimeter zones and one core zone without any critical zones. Future research can focus on a multi-storey model with more core zones and some critical zones to investigate how much energy can be saved on a big scale and how fan energy can play a role in the total HVAC energy consumption when the building is complex. Additionally, the results of this paper are only from the simulation, and the EnergyPlus model did not undergo calibration processes. It is recommended that future work compare the simulated results with measured data from real buildings and adjust the model parameters accordingly. Furthermore, this paper did not investigate the impact of the proposed faults on energy consumption through actual building operation data. We recommend that future work should present the impact of faults on energy consumption through actual building operation data by collecting the building management system (BMS) data and cleaning and aggregating it into appropriate time intervals to align with EnergyPlus simulation results. Then, the energy consumption patterns and performance metrics can be compared under normal operation and during fault conditions to identify significant deviations caused by each fault and assess their implications for building operation and energy efficiency. Furthermore, in this paper, the SAT and DSP setpoints were kept constant when studying each other’s and MOP’s logic robustness. Future work can investigate the impact of zone-level faults when all G36 sequences of operation are employed in the building BAS. Moreover, future work can explore the impact of the injection of faults in different zones. For example, a faulty north-facing zone may have a different effect on EUI than a faulty south-facing zone. Finally, it would be worthwhile to have a future study on the costs of implementing and maintaining G36 strategies as they require more complex algorithms at the BAS level, often misunderstood or improperly employed by facilities personnel.

## Conclusion

Customized sequences of operation were implemented in the EMS application of the BPS tool EnergyPlus to investigate their energy efficiency and robustness to hardware faults at the zone level for single-duct multi-zone VAV AHU systems. First, different scenarios that are commonly used in the building industry or proposed by researchers for three vulnerable control logics to zone-level faults: SAT setpoint, DSP setpoint and MOP temperature-based control, were defined in the EMS. Then, the most common faults at the zone level with different temperature and pressure impacts on the zone were integrated into one of the zones. The results revealed that in both fault-free zones and faulty zone situations, the T&R reset logic that can successfully identify the rogue zone is the most energy-efficient and fault-tolerant scenario for the SAT setpoint and DSP setpoint logic. Moreover, results demonstrated that when a fault causes overcooling in a zone, the zone is not identified as a rogue zone in the SAT setpoint reset logic. Since single-duct multi-zone VAV AHU systems are intended for cooling, heating requests are not generated even though the zone may not be thermally comfortable. Similarly, a fault that causes the zone to appear as a well-fed zone does not generate pressure-increase requests, so the zone is not considered a rogue zone in DSP setpoint reset logic, although it may cause thermal discomfort.

Implementing G36 sequences of operation for all AHU control loops will result in the identification of most of the rogue zones, and a zone not identified as a rogue zone in one reset logic may be considered a rogue zone in another reset logic. For example, a zone with a stuck VAV damper in the fully open position, which does not generate heating requests, is not considered a rogue zone in the SAT setpoint reset logic but will be identified as a rogue zone in the DSP setpoint reset logic as it constantly generates pressure increase requests. Moreover, aside from alarms generated when rogue zones are identified, G36 also defines high- and low-temperature alarms triggered when zones deviate 2°C or 3°C from their setpoint temperature, facilitating the identification of faulty zones that do not fall under the category of rogue zones but still contribute to thermal discomfort. Finally, for the MOP logic, the average temperature–based control scenario significantly reduces the activation of the night cycle compared to the min/max temperature–based control logic. The identification of rogue zones in MOP logic is by the zone high- and low-temperature alarms, which, as per G36, cannot be suppressed in setup, setback or unoccupied modes to avoid excessive pull-down or pick-up loads and even pipes freezing.
